# Health‐Related Behaviours in Octogenarians and Nonagenarians with Robust Cognitive Longevity: Progress from the SuperAging Research Initiative

**DOI:** 10.1002/alz70860_105769

**Published:** 2025-12-23

**Authors:** Angela C. Roberts, Karen Van Ooteghem, Ivan Culum, Kit B. Beyer, Bill McIlroy, Andrew Lim, Richard H. Swartz, Desmond O. Oklikah, Emily Narayan, Elizabeth Finger, Amanda Cook Maher, Felicia C. Goldstein, Adam Martersteck, Ozioma C. Okonkwo, Rhiana Schafer, Emily J Rogalski, SuperAging Research Initiative

**Affiliations:** ^1^ Canadian Centre for Activity and Aging, London, ON, Canada; ^2^ Western University, London, ON, Canada; ^3^ University of Waterloo, Waterloo, ON, Canada; ^4^ Sunnybrook Research Institute, Toronto, ON, Canada; ^5^ Temerty Faculty of Medicine, University of Toronto, Toronto, ON, Canada; ^6^ University of Toronto, Toronto, ON, Canada; ^7^ Lawson Health Research Institutes, London, ON, Canada; ^8^ University of Michigan, Ann Arbor, MI, USA; ^9^ Emory University School of Medicine, Atlanta, GA, USA; ^10^ University of Chicago, Chicago, IL, USA; ^11^ University of Wisconsin‐Madison School of Medicine and Public Health, Madison, WI, USA; ^12^ Healthy Aging & Alzheimer's Research Care (HAARC) Center, University of Chicago, Chicago, IL, USA

## Abstract

**Background:**

SuperAgers—individuals age 80+ with episodic memory performance at least as good as those 20‐30 years younger—provide a unique perspective on cognitive resilience and resistance in aging. The SuperAging Research Initiative (SRI), spearheaded by The University of Chicago and involving multiple academic partners, investigates factors underpinning robust cognitive aging. One key SRI project leverages a fully remote data collection paradigm to 1) discern activity patterns that characterize SuperAgers and 2) explore the 'complexity hypothesis in aging'—whether dynamic physiological responsiveness is a hallmark of exceptional cognitive aging.

**Method:**

In a fully remote data collection protocol, participants don wearable sensors, including an ECG sensor (chest) and two inertial measurement units (wrist, ankle), for a 10–12‐day period of continuous data collection whilst performing their usual daily activities. The protocol includes a virtual orientation and periodic check‐ins to ensure wear compliance and provide technical support. Structured sensor‐wear breaks facilitate protocol compliance and acceptance. Recruitment strategies include a structured communication plan and updates with SRI sites, coordinator education, ongoing updates to foster SRI engagement, and a warm hand‐off protocol to facilitate communication.

**Result:**

To date, 154 persons (Mean age = 83.3 years; 94 women), > 70% of the new SRI core study cohort, have enrolled. Reasons for ineligibility or non‐enrollment include dermatological contraindications, ‘busy’ lifestyles, and perceived participation burden. Compliance with the sensor wear protocol has been consistently robust, with >90% of usable expected wear data for the protocol duration. Limited data loss due to sensor non‐wear and/or sensor failure demonstrates high data quality. Participants demonstrate high independence, with study partner assistance required in 5.2% (*N* = 8) of cases. Withdrawals after starting data collection have been minimal (*N* = 3, 2.0%) and primarily attributed to skin irritation due to undisclosed dermatological contraindications or discomfort when wearing sensors at night. Thirteen (8.44%) adverse events (nonserious) have been reported, all involving minor skin irritation or bruising.

**Conclusion:**

Wearable technologies are feasible for remotely assessing the daily activities of octogenarians and nonagenarians with high fidelity. Insights from this study may influence preventive intervention strategies against age‐associated neurological diseases and support the enhancement of cognitive longevity.

